# Galli gigeriae endothelium corneum: its intestinal barrier protective activity in vitro and chemical composition

**DOI:** 10.1186/s13020-021-00432-3

**Published:** 2021-02-16

**Authors:** Shanshan Li, Meng Zheng, Zhentang Zhang, Hengying Peng, Wenling Dai, Jihua Liu

**Affiliations:** 1 Jiangsu Key Laboratory of TCM Evaluation and Translational Research, School of Traditional Chinese Pharmacy, China Pharmaceutical University, Nanjing, 211198 People’s Republic of China; 2 State Key Laboratory of Natural Medicines, China Pharmaceutical University, Nanjing, 211198 People’s Republic of China

**Keywords:** Galli gigeriae endothelium corneum, HPLC-QTOF–MS/MS, Identification, Anti-inflammatory, Wound healing, Gastrointestinal barrier

## Abstract

**Background:**

Galli gigeriae endothelium corneum (GGEC) has been effectively used for centuries for the treatment of functional dyspepsia (FD) in clinical practice in Asian countries. However, its potential mechanism and chemical composition remains undertermined.

**Methods:**

In this study, the chemical profile of GGEC ethyl acetate extract (EAE) was evaluated by HPLC-Q-TOF–MS/MS. The effects of EAE on intestinal barrier function and inflammation were investigated in IEC-6 cells and RAW264.7 cells.

**Results:**

The results showed that 33 compounds were tentatively identified, including 12 soy isoflavones, 7 bile acids for the first time in EAE. EAE significantly reinforced intestinal barrier function via increasing the tight junction protein levels of ZO-1 and Occludin, reducing the mRNA expression levels of interleukin (IL)-1β and IL-6 in tumor necrosis factor alpha (TNF-α)-challenged IEC-6 cells. The scratch wound assay showed that EAE accelerated wound healing of IEC-6 cells. EAE evidently reduced the level of NO in a dose-dependent manner with an IC_50_ value of 18.12 μg/mL, and the mRNA expression of TNF-α, IL-1β, IL-6, iNOS and COX-2 in LPS-treated RAW264.7 cells.

**Conclusion:**

This study revealed the intestinal barrier protective effects and chemical profile of GGEC, and the results indicated that GGEC strengthened the intestinal barrier by up-regulating protein expression of tight junctions and limiting inflammatory responses.

## Background

Intestinal epithelial cells (IECs) play a vital role in digestion and nutrient absorption and prevent harmful agents from entering the body acting as a physical barrier to maintain intestinal homeostasis [1]. The integrity of the intestinal barrier is closely related to intestinal health, and it is regulated by the interaction of various barrier components, such as the mucous layer, antibacterial peptides, and tight junctions (TJs) [2]. Therefore, maintaining a proper TJ expression level is widely considered as an effective therapeutics target for the treatment of intestinal diseases [3].

The intestinal epithelium can be injured by several factors such as toxic luminal substances, inflammation, and oxidative stress [4]. After injury, the balance between anti- and pro-inflammatory cytokines becomes disrupted and pro-inflammatory cytokine secretion is increased [5]. Recent studies have indicated that inhibition of cytokines induces increase in intestinal permeability, resulting in an important protective effect against intestinal epithelial damage and intestinal inflammation [6]. Therefore, it is necessary to improve gut health through suppressing unnecessary inflammatory responses [7].

Galli gigeriae endothelium corneum (GGEC), the dried inner wall of the *Gallus gallus* domesticus Brisson as a chicken by-product, is described in the Chinese pharmacopoeia as a well-known traditional Chinese drug and an edible food [8]. GGEC is widely used in Asian countries in clinical practice for the treatment of diarrhea, dyspepsia, infantile malnutrition, and mammary gland proliferation, especially in the treatment of children with indigestion [9]. In recent years, there have been studies on the chemical composition of GGEC, which have mainly focused on macromolecules such as proteins and polysaccharides [10]. However, information on the micro-molecules of GGEC is still limited.

Studies have revealed that injured intestinal barrier [11] and over-inflammation are the characteristics of gastrointestinal disorders [12]. Therefore, here, we explored the effects of GGEC on the intestinal epithelial barrier as well as its chemical composition aiming to provide a theoretical basis for further research on the clinical use of GGEC for improving gut health.

## Materials and methods

### Chemicals and reagents

Rat intestinal epithelial (IEC-6) cells and RAW 264.7 murine macrophage cell line (ATCC, USA) were cultured in DMEM supplemented with 10% fetal bovine serum (FBS), 100 units/mL penicillin, 100 μg/mL streptomycin and 0.1 unit/mL human insulin (for IEC-6 cells) and maintained in a carbon dioxide incubator (Thermo Fisher Scientific, USA) with a humidified atmosphere of 95% air and 5% CO_2_ at 37 °C.

### Preparation of samples

GGEC was collected from the Traditional Chinese Medicine Market (Bozhou, China), and identified and retained at Jiangsu Key Laboratory of TCM Evaluation and Translational Research, China Pharmaceutical University (Nanjing, China). GGEC ethyl acetate extract (EAE) was prepared as follows. The powered GGEC (5 kg) was extracted twice with 70% ethanol (v/v) in a hot water bath, each time for 1 h. Ethanol was removed with a rotary evaporator and part of the 70% extract was suspended in water and extracted successively with petroleum ether and ethyl acetate, respectively. After evaporation of the solvent in vacuum, the ethyl acetate fraction (EAE, 50 g) was obtained. The EAE was dissolved in dimethyl sulfoxide (DMSO) for used as a stock solution (10 mg/mL), which was diluted in the medium and added to the cells at different concentrations (The final concentration of DMSO in medium was no more than 0.1%).

### Chemical identification

#### HPLC-QTOF–MS/MS analysis

The chemical analysis of the EAE was performed on an Agilent 1260 series HPLC system (Agilent Technologies, USA). Sample separation was achieved on an Agilent ZORBAX SB-C_18_ column (4.6 mm × 250 mm, 5 μm) with a constant flow rate of 1.0 mL/min at 30 °C. The mobile phase was composed of water (0.1% formic acid, A) and acetonitrile (B) using gradient elution. The gradients were operated as follows: 0–60 min, 10–90% B; 60–62 min, 90–10% B; 62–70 min, 10% B. The sample volume injected was set at 10 μL. The peaks were monitored at 254 and 280 nm.

An Agilent 6530 Q-TOF mass spectrometer (Agilent Technologies, USA) equipped with an electrospray ionization (ESI) source was used to perform the MS analysis. The acquisition parameters were as follows: drying gas (N2) flow rate, 10.0 L/min; drying gas temperature, 350 °C; nebulizer, 35 psig; capillary, 3500 V; OCTRFV, 750 V; and fragmentor voltage, 120 V. The mass range was recorded from *m*/*z* 50 to 1500 in positive and negative modes with collision energy (CE) from 10 to 50 eV. Peaks were detected by positive and negative ionization mode of MS and MS/MS detection. All operations, data acquisition and analysis were controlled by Agilent Mass Hunter Workstation software version B.07.00.

### Compounds identification

The obtained HPLC-QTOF–MS/MS data were interpreted with the authentic reference standards or with its structural analogues, analyzed in identical experimental conditions to compare their chromatographic and mass spectral profiles and the literature [13, 14].

### Assessment of intestinal barrier function

The intestinal barrier function was evaluated as previously reported [15]. Cells were incubated with EAE (0.1, 0.03, and 0.01 mg/mL) for 1 h, then TNF-α (50 ng/mL) was added to the apical side for 24 h. The transepithelial electrical resistance (TEER) was monitored using a Millicell-ERS volt-ohmmeter (Millipore, USA). FD-4 (Sigma-Aldrich) with a final concentration of 1 mg/mL dissolved in Krebs was added to the apical side of the insets and 600 μL Krebs was added to the basolateral side [16]. The basolateral medium was taken after 1 h of incubation. The diffused fluorescent tracer was then measured by fluorometry (excitation, 485 nm; emission, 528 nm). The data are presented as a percentage of the control group [17].

### Western blotting analysis

Cells were rinsed with ice-cold PBS buffer thrice and lysed with RIPA lysis buffercontaining 1 mM PMSF on ice for 30 min. The cell debris was subsequently removed by centrifugation at 12000*g* for 10 min at 4 °C. Then the supernatant was collected for analysis by adding using 5 × SDS loading buffer containing 7% β-mercaptoethanol. Equal amounts of protein samples were separated by 10% (v/v) SDS-PAGE and transferred onto PVDF membranes (0.22 μm, Millipore, USA). After blocking with 5% BSA at room temperature for 2 h, the membranes were incubated with the corresponding primary anti-ZO-1 (1:1000; Proteintech, USA), anti-GAPDH (1:8000; Sigma, USA) and anti-Occludin (1:1000; Proteintech, USA) antibodies at 4 °C overnight. The membranes were then incubated with HRP-conjugated anti-rabbit IgG (1:4000; Cell Signaling Technology, USA) secondary antibodies for 2 h at room temperature, and the immunoreactive protein bands were visualized using ECL detection reagents (Bio-Rad, USA). The intensity of protein bands was quantitated using an Image Lab analysis software and normalized to GAPDH.

### Anti-inflammatory effects

#### Cell viability and nitric oxide (NO) inhibition assay

RAW264.7 cells were plated into a 96-well plate at a density of 1 × 10^4^/well in 200 μL of DMEM medium and incubated overnight. Then the medium was replaced with various concentrations of EAE including 0.1, 0.03, and 0.01 mg/mL and incubated for another 24 h. Cell culture media were collected, following the measurement of the nitrite level using Griess reaction assay. Cytotoxicity of EAE was evaluated by MTT assay. The percentage of cell viability was calculated as a percentage of the control. The IC_50_ of NO inhibition rate was calculated using GraphPad (GraphPad Software, San Diego, CA, USA).

### Real-time quantitative reverse transcription polymerase chain reaction (qRT-PCR)

RAW 264.7 cells /IEC-6 cells were cultured at 1 × 10^6^ cells/well in 6-well plates and pretreated in the absence or presence (0.1, 0.03, and 0.01 mg/mL) of EAE for 1 h, prior to being untreated or stimulated with LPS (1 μg/mL) or TNF-α (50 ng/mL) for 24 h. The cells were harvested, and total RNA was extracted using TRI reagent solution (TransGen Biotech,Beijing, China). The following primers were used for qPCR: GAPDH, forward, 5′-CAGGGCTGCCTTCTCTTGTG-3′ and reverse, 5′-GATGGTGATGGGTTTCCCGT-3′; IL-6, forward, 5′-AATCTGCTCTGGTCTTCTGG-3′ and reverse: 5′-GATGAGTTGGATGGTCTTGG-3′; IL-1β, forward, 5′-CCAGGATGAGGACCCAAGCA-3′ and reverse, 5′-TCCCGACCATTGCTGTTTCC-3′; GAPDH (mice), forward, 5′-CAGTGGCAAAGTGGAGATTG-3′ and reverse, 5′-GTTGTCATGGATGACCTTGG-3′; IL-6 (mice), forward, 5′-GCTACCTGGAGTACATGAAG-3′ and reverse, 5′-CTGTGACTCCAGCTTATCTG-3′; IL-1β (mice), forward, 5′-ATGAGGACATGAGCACCTTC-3′ and reverse, 5′-CATTGAGGTGGAGAGCTTTC-3′; TNF-α(mice), forward, 5′-CTCAGATCATCTTCTCAAAATTCGAGTGACA-3′ and reverse, 5′-CTTCACAGAGCAATGACTCCAAAGT-3′; iNOS (mice), forward, 5′-CTCAGATCATCTTCTCAAAATTCGAGTGACA-3′ and reverse, 5′-CTTCACAGAGCAATGACTCCAAAGT-3′; COX-2 (mice), forward: 5′-ATGGTCAGTAGACTTTTACA-3′ and reverse: 5′-GGAGAGACTATCAAGATAGT-3’.

### Wound healing assay

IEC-6 cells were seeded into six-well plates at a density of 5 × 10^5^ cells/well, and were cultured in fresh cultured media to 90% confluence. After being washed with PBS, the medium was replaced with DMEM-1% FBS. EAE (0.1, 0.03, and 0.01 mg/mL) was added to the corresponding chambers. The cell migration images were photographed at 0 and 8 h following scraping [5]. The wound scratches were measured using Image J software. Three to four different fields were visualized and photographed with an optical microscope at 100 × magnification (Olympus IX53, Tokyo, Japan). The migration rates were calculated using the following formula: [(the initial width) − (the width after 8 h of culturing)]/(the initial width) × 100%.

### Statistical analysis

Values are presented as mean ± SD. Differences among groups were analyzed by one-way ANOVA with Dunnett’s test using GraphPad 5.0 software. The value of *p* < 0.05 was considered statistically significant.

## Results

### Identification of constituents in EAE of GGEC

**Fig. 1:**
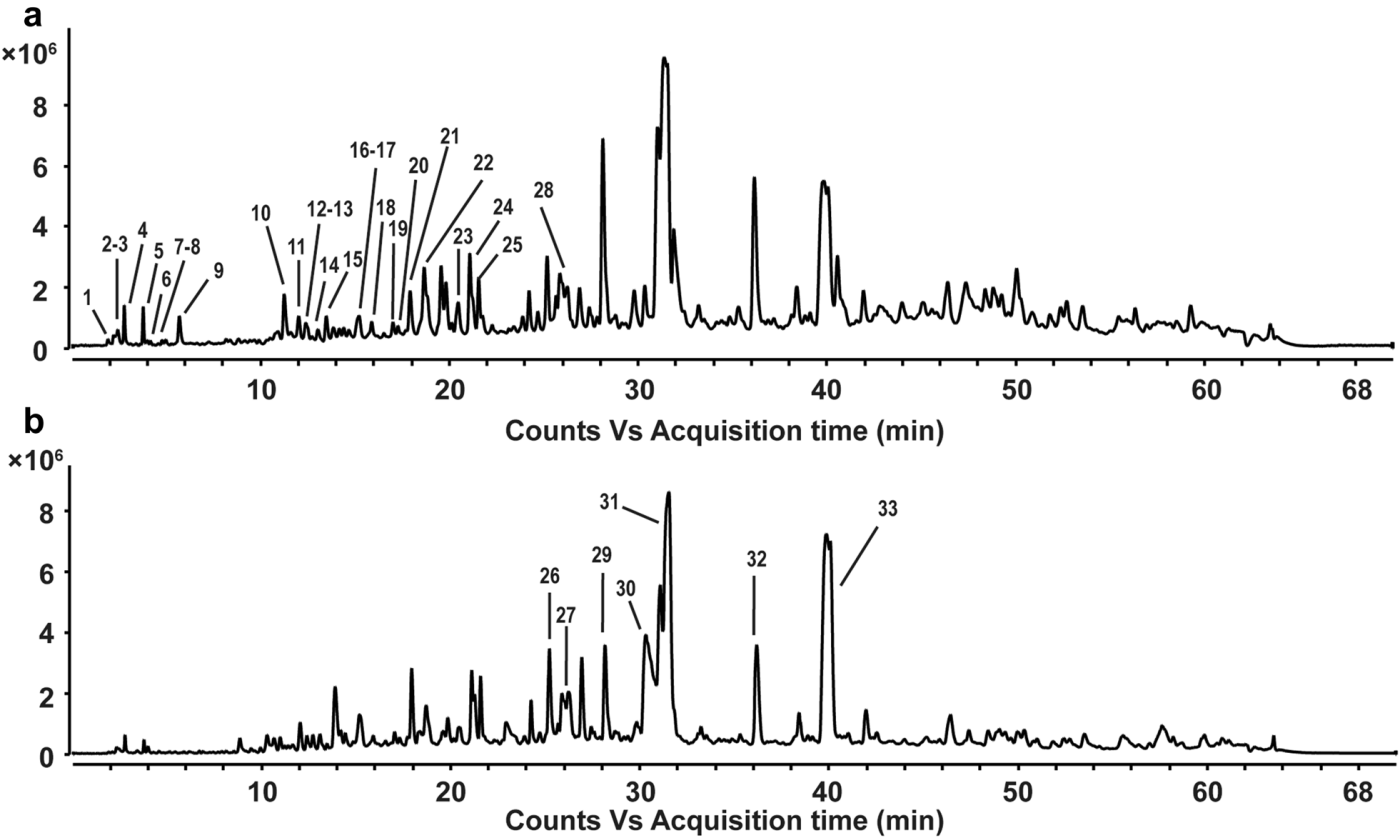
Total ion chromatograms (TIC) in the positive-ion mode (**a**) and negative-ion mode (**b**) from HPLC-QTOF–MS/MS of EAE. EAE: Galli gigeriae endothelium corneum ethyl acetate extract

A total of 33 compounds were identified from EAE by HPLC-Q-TOF–MS/MS in the positive/ negative ion mode (Fig. 1). The retention time and mass spectrometry information of each chemical constituent of GGEC were detected as shown in Table 1. Thirty-three compounds including 12 soy isoflavones, 7 bile acids, 4 nucleic bases and nucleosides and 10 other compounds were identified. Except the amino acids, these compounds were reported here in GGEC for the first time.

**Table 1:** The chromatographic and mass spectrometric data of galli gigeriae endothelium corneum ethyl acetate extract using HPLC-QTOF–MS/MS

	RT	Precusor ion (amu)M	Selective ion	*m*/*z*		Mass error (ppm)	Formula	Observed diagnostic CID fragment ions	Identification
				Measured	Calculated				
1	2.28	113.0589	[M + H]^+^	114.0664	114.0662	− 1.87	C_4_H_7_N_3_O	72.0562	Creatinine [19]
2	2.42	135.0538	[M + H]^+^	136.0611	136.0604	− 3.45	C_5_H_5_N_5_	119.0342, 92.0241, 65.0132	Adenine [19]
3	2.52	151.0494	[M + H]^+^	152.0559	152.0567	1.78	C_5_H_5_N_5_O	135.0291, 110.0347, 80.0259	Guanine [20]
4	2.77	136.0377	[M + H]^+^	137.0450	137.0458	2.79	C_5_H_4_N_4_O	119.0345, 110.0354, 94.0384	Hypoxanthine [20]
5	3.77	267.0952	[M + H]^+^	268.1025	268.1040	3.73	C_10_H_13_N_5_O_4_	136.0618	Adenosine [20]
6	3.92	122.048	[M + H]^+^	123.057	123.0553	− 1.02	C_6_H_6_N_2_O	109.0464, 80.0465, 53.0366	Niacinamide [19]
7	4.96	168.0896	[M + H]^+^	169.0969	169.0972	− 0.27	C_8_H_12_N_2_O_2_	98.0600, 70.0656	Pyridoxamine [19]
8	5.18	131.0946	[M + H]^+^	132.1014	132.1019	1.85	C_6_H_13_NO_2_	86.0965, 69.0701, 57.0580, 44.0500	Leucine/isoleucine [21]
9	5.97	129.0574	[M + H]^+^	130.0647	130.0651	3.3	C_9_H_7_N	103.0546, 77.0385	Isoquinoline [20]
10	11.16	173.1052	[M + H]^+^	174.1146	174.1125	− 2.31	C_8_H_15_NO_3_	90.0492, 57.0671	Acly-glycine [21]
11	12.00	416.1075	[M + H]^+^	417.1148	417.1180	0.71	C_21_H_20_O_9_	255.0627	Daidzin [23]
12	12.36	446.1176	[M + H]^+^	447.1249	447.1286	2.3	C_22_H_22_O_10_	285.0751	Glycitin [23]
13	12.37	446.0817	[M + H]^+^	447.0890	447.0902	3.3	C_21_H_18_O_11_	271.0591	Genistein-*O*-glucuronide [24]
14	13.02	276.1090	[M + H]^+^	277.1163	277.1183	0.3	C_14_H_16_N_2_O_4_	231.1117, 186.0894	*N*-lactoyl-tryptophan [21]
15	13.50	210.1348	[M + H]^+^	211.1421	211.1441	2.4	C_11_H_18_N_2_O_2_	138.1272, 70.0655	*L*,*L*-Cyclo(leucylprolyl) [22]
16	15.03	502.1074	[M + H]^+^	503.1147	503.1184	0.01	C_24_H_22_O_12_	255.0631	6″-*O*-Malonyldaidzin [24]
17	15.20	432.103	[M + H]^+^	433.1103	433.1129	2.68	C_21_H_20_O_10_	271.0581	Genistin [23]
18	15.74	175.0621	[M + H]^+^	176.0730	176.0706	− 1.11	C_10_H_9_NO_2_	130.0588	Indoleacetic acid [30]
19	17.02	458.1188	[M + H]^+^	459.1261	459.1286	2.34	C_23_H_22_O_10_	255.0634	6″-*O*-Acetyldaidzin [24]
20	17.28	488.1288	[M + H]^+^	489.1361	489.1391	3.15	C_24_H_24_O_11_	285.0737	6″-*O*-Acetylglycitin [24]
21	17.91	518.1029	[M + H]^+^	519.1102	519.1133	− 0.55	C_24_H_22_O_13_	271.0584	6″-Malnoylgenistin [24]
22	18.67	145.0527	[M + H]^+^	146.0602	146.0621	0.28	C_9_H_7_NO	91.0547, 77.0392	1*H*-Indole-3-carboxaldehyde [31]
23	20.35	474.1146	[M + H]^+^	475.1219	475.1235	3.35	C_23_H_22_O_11_	271.0578	6″-*O*-Acetylgenistin [24]
24	21.25	254.0563	[M + H]^+^	255.0636	255.0652	3.24	C_15_H_10_O_4_	237.0527, 137.0223	Daidzein [23]
25	21.76	284.0666	[M + H]^+^	285.0739	285.0757	2.51	C_16_H_12_O_5_	270.0501, 242.0548	Glycitein [23]
26	25.21	515.2909	[M + H]^−^	514.2836	514.2844	1.55	C_26_H_45_NO_7_S	353.2490, 124.0074, 106.9810, 80.0027	Taurocholic acid [28, 29]
27	26.15	465.3094	[M + H]^−^	464.3021	464.3018	− 0.73	C_26_H_43_NO_6_	435.3073, 402.2974, 74.0251	Glycocholic acid [27]
28	26.72	270.0511	[M + H]^+^	271.0584	271.0601	3.29	C_15_H_10_O_5_	153.0171	Genistein [23]
29	28.14	406.2692	[M + H]^−^	405.2619	405.2636	3.76	C_24_H_38_O_5_	389.2649, 371.2541, 353.2437	Oxocholic acid [26]
30	30.31	499.2969	[M + H]^−^	498.2896	498.2895	− 0.24	C_26_H_45_NO_6_S	480.2758, 124.0074, 106.9810, 80.0027	Taurochenodeoxycholic acid [28]
31	31.23	408.2861	[M + H]^−^	407.2803	407.2788	3.67	C_24_H_40_O_5_	389.2641, 343.2629, 289.2168	Cholic acid [25, 26]
32	32.09	449.3138	[M + H]^−^	448.3065	448.3068	0.77	C_26_H_43_NO_5_	405.2652, 386.3051, 330.2422, 74.0251	Glycochenodeoxycholic acid [27]
34	39.88	392.2925	[M + H]^−^	391.2852	391.2854	0.47	C_24_H_40_O_4_	373.2694	Chenodeoxycholic acid [25, 26]

#### Nucleic bases and nucleosides

Nucleobases are nitrogen containing biological compounds linked to a sugar within nucleosides making up the basic building blocks of DNA and RNA. Nucleobases and nucleosides play important roles in the process of lives [18]. The most common fragmentation process for nucleic bases is the loss of H_2_O and/or NH_3_, followed by the removal of HCN or CO. Compound 2 was identified as adenine with *m*/*z*^+^ 136.0611 and diagnostic fragmentation *m*/*z*^+^ 119.0342 [M + H-NH3]^+^. Similarly, compound 5 had *m*/*z*^+^ 268.1025 with C_10_H_13_N_5_O_4_ was adenosine [19]. The observed *m*/*z*^+^ 152.0559 of compound 3 with formula C_5_H_5_N_5_O was identified as guanine [20]. Compound 4 was identified as hypoxanthine with formula C_5_H_4_N_4_O as it produced fragment ions at *m*/*z*^+^ 119.0345 and 110.0354.

#### Amino acids and peptides

Although various free amino acids can be detected in GGEC water extract, in this part only few of them can be detected maybe due to their hydrophobicity. Compound 8 displayed a molecule at *m*/*z*^+^ 132.1014 with the molecular formula C_6_H_13_NO_2_, which was presumed tentatively as leucine or isoleucine [21]. In addition, a cyclodipeptide with the molecular formula C_11_H_18_N_2_O_2_ was presumed as *L*,*L*-cyclo(leucylprolyl) [22].

#### Isoflavones

Isoflavones were firstly identified from GGEC. As many as 12 isoflavones were identified by HPLC-Q-TOF–MS/MS in this study. Among them, compounds 24, 25, 28 showed mass spectral responses for parent ions that lead to the formation of isoflavone aglycone fragment ions at *m*/*z*^+^ 255.0636 for daidzein, *m*/*z*^+^ 285.0739 for glycitein, and *m*/*z*^+^ 271.0584 for genistein [23]. Compounds 11, 12, 17 were unambiguously identified as daidzin (*m*/*z*^+^ 417.1148), glycitin (*m*/*z*^+^ 447.1249), genistin (*m*/*z*^+^ 433.1103) with observed the loss of 162.

The remaining compounds could be tentatively assigned according to the literature from the accurate mass data [24]. Compounds 16 and 19 had *m*/*z*^+^ 503.1184 and 459.1261, and the chemical formula were C_24_H_22_O_12_ and C_23_H_22_O_10_, respectively. They were presumed to be 6″-*O*-malonyldaidzin and 6″-*O*-acetyldaidzin according to the fragmentation patterns. Similarly, compounds 13, 21 and 23 had *m*/*z*^+^ 447.0922, 519.1133 and 475.1235 with the formula C_21_H_18_O_11_, C_24_H_22_O_13_ and C_23_H_22_O_11_ were presumed as genistein-*O*-glucuronide, 6″-malnoylgenistin and 6″-*O*-acetylgenistin, respectively. Compound 20 was presumed to be 6″-*O*-acetylglycitin as with its formula C_24_H_24_O_11_. These six compounds were deduced to be generated based on the nuclei.

#### Bile acids

ESI–MS/MS spectra in both negative and positive ion modes were examined in this study. According to the fragmentation patterns in negative mode, the BAs could be classified into three groups, namely free Bas, taurine- and glycine-conjugated Bas. As shown in Table 1, free BAs were obtained via the neutral losses of H_2_O and CO_2_ molecules [25]. Compound 31, compound 33 and compound 29 had *m*/*z*^−^ 407.2794, 391.2839 and 405.2619 and the formula were C_24_H_40_O_5_, C_24_H_40_O_4_ and C_24_H_38_O_5_, respectively. They were cholic acid (CA), chenodeoxycholic acid (CDCA) and oxocholic acid according to the reference and literature [26]. According to the literature [27], the detection of a product ion at *m*/*z* 74 strongly suggested the existence of a glycine moiety at the side-chain terminus. In the case of taurine conjugates, three productions at *m*/*z* 80, *m*/*z*107, and *m*/*z* 124 invariably appeared in the product ion spectrum [28]. Compound 26 and compound 30 with *m*/*z*^−^ 514.2865 and 498.2888 were tended to be taurocholic acid (TCA) and taurochenodesoxychlic acid (TCDCA) and the chemical formula were C_26_H_45_NO_7_S and C_26_H_45_NO_6_S, respectively [29]. Compound 27 and compound 32 with *m*/*z*^−^ 464.3010 and 448.3065 with formula C_26_H_43_NO_6_ and C_26_H_43_NO_5_ were presumed as glycocholic acid (GCA) and glycochenodeoxycholic acid (GCDCA).

#### Other compounds

Compound 18 displayed a protonated molecule at *m*/*z* 176.0730 [M + H]^+^ with the molecular formula C_10_H_9_NO_2_, which is consistent with the literature [30], and presumed to be Indoleacetic acid. Compound 22 with formula C_9_H_7_NO at *m*/*z*^+^ 146.0602 was presumed as 1H-indole-3-carboxaldehyde [31].

### EAE alleviated the intestinal epithelial barrier dysfunction induced by TNF-α

**Fig. 2:**
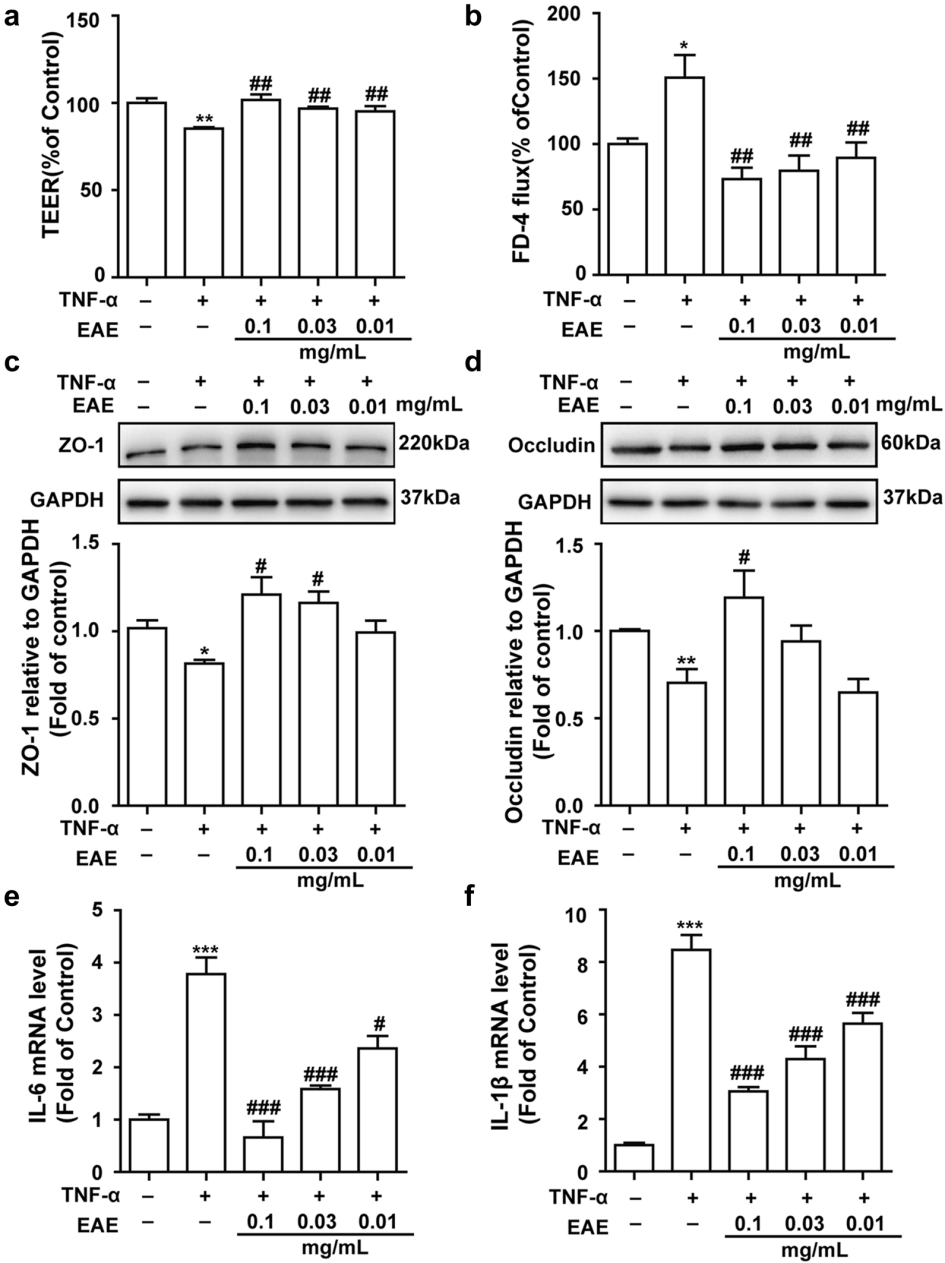
EAE exerted protective effects on TNF-α-treated IEC-6 cells. When cells reached confluence in the transwell system, Cells were pretreated with EAE (0.1, 0.03, and 0.01 mg/mL) for 1 h, followed by TNF-α (50 ng/mL) stimulation for 24 h. **a** TEER values were shown as percentage relative to the control. **b** Permeability of FD-4 in TNF-α-treated IEC-6 cell. IEC-6 cells were pretreated with EAE (0.1, 0.03, and 0.01 mg/mL) for 1 h, followed by TNF-α (50 ng/mL) stimulation for 24 h. The relative ratios of ZO-1/GAPDH and Occludin/GAPDH were calculated based on the densities of bands on Western blots. The protein expression levels of ZO-1 (**c**) and occludin (**d**) were increased in TNF-α-treated IEC-6 cells while pretreated with EAE. EAE decreased the mRNA expression levels of IL-6 (**e**) and IL-1β (**f**) in TNF-α-stimulated IEC-6 cells. Mean ± SD (n = 3 independent experiments). *p < 0.05, **p < 0.01 vs. the control group. #*p* < 0.05, ##*p* < 0.01 vs. the TNF-α-stimulated group. EAE: galli gigeriae endothelium corneum ethyl acetate extract

The effect of EAE was examined on the paracellular permeability of IEC-6 cells treated with TNF-α. As displayed in Fig. 2a, the TEER of the TNF-α-damaged IEC-6 cell monolayer reduced obviously compared with the control group, showing that TNF-α upregulated the paracellular permeability of the IEC-6 cell monolayer. By contrast, this decreased TEER was obviously inhibited by co-treatment with EAE. As shown in Fig. 2b, TNF-α increased the FD-4 flux in the IEC-6 cell monolayer, showing that this inflammatory cytokine increased the paracellular permeability of the IEC-6 cell monolayer. However, this increased FD-4 flux was obviously inhibited by co-administration of EAE. These findings showed that EAE alleviated the TNF-α-induced disorder of the intestinal epithelial barrier function.

### EAE prevented the TNF-α-induced down-regulated expression of TJ proteins ZO-1 and occludin

In the present study, western blot analysis of ZO-1 and occludin were carried out to investigate the effect of EAE on epithelial barrier-relevant TJ protein expression. As shown in Fig. 2c, d, our results indicated that TNF-α challenge caused an obvious decrease of ZO-1 and occludin protein expressions. However, treatment of the IEC-6 cells with EAE alleviated the TNF-α-induced ZO-1 and occludin protein decline.

### EAE suppressed the pro-inflammatory cytokines secretion from TNF-α-induced IEC-6 cells

TNF-α was used to stimulate pro-inflammatory cytokine secretion in IEC-6 cells to imitate its elevation in some intestinal diseases. As shown in Fig. 2e, f, our results indicated that the levels of IL-6 and IL-1β in IEC-6 cells was obviously increased after TNF-α (50 ng/mL) stimulation. However, treatment of the IEC-6 cells with EAE (0.1–0.01 mg/mL) for 24 h caused an obvious decrease in these pro-inflammatory cytokine levels in a dose-dependent manner. These results showed that EAE can regulate the TNF-α-induced secretion of pro-inflammatory cytokines.

### Anti-inflammatory effects on RAW264.7 cells

**Fig. 3:**
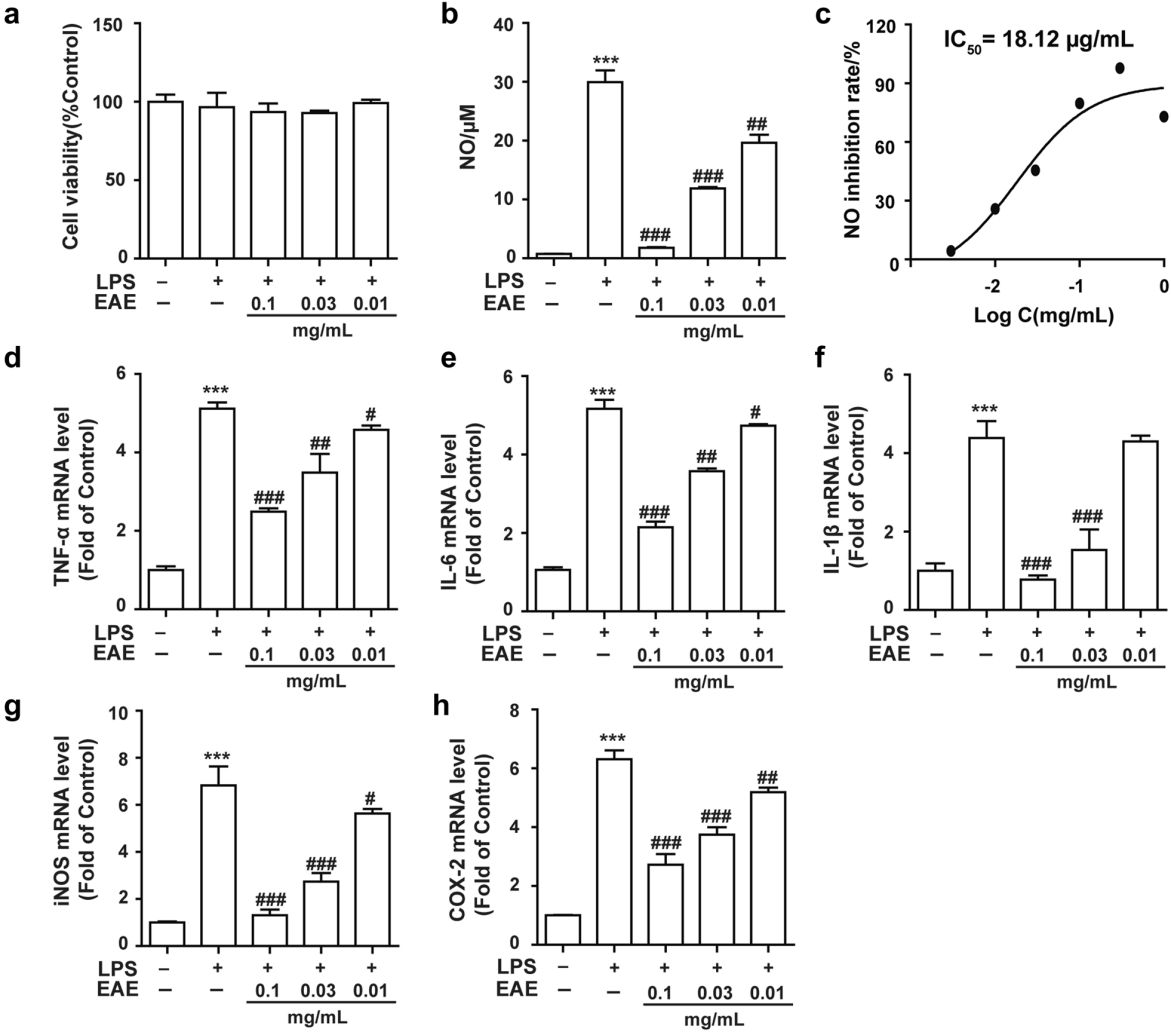
The anti-inflammatory effects of EAE on RAW264.7 cells. **a** The cytotoxic effect of EAE on macrophages viability in LPS-stimulated RAW264.7 cells. **b** The effect of EAE on nitric oxide (NO) production in LPS- stimulated RAW264.7 cells. **c** The IC_50_ for inhibition of LPS-induced NO. The effect of EAE on the mRNA expression level of TNF-α (**d**), IL-6 (**e**), IL-1β (F), iNOS (**g**) and COX-2 (H) in LPS-stimulated RAW 264.7 cells. RAW264.7 cells were stimulated with or without 1 μg/mL of LPS for 24 h. Mean ± SD (n = 3 independent experiments). ***p < 0.001 versus control group; #*p* < 0.05, ##*p* < 0.01 and ###*p* < 0.001 versus LPS-only treatment group. EAE: galli gigeriae endothelium Corneum ethyl acetate extract

Our results suggested that it had no obvious cytotoxic effects on the RAW264.7 cell when the concentration was lower than 0.1 mg/mL (Fig. 3a). Then the cell was treated with LPS (1 μg/mL) with/without EAE for 24 h. As in Fig. 3b, the results showed that EAE significantly inhibited LPS-stimulated NO production along with the extract concentrations with IC_50_ of 18.12 μg/mL (Fig. 3c).

Furthermore, the mRNA expression levels of inflammatory mediators (iNOS and COX-2) and pro-inflammatory cytokines (TNF-α, IL-6 and IL-1β) were investigated by qRT-PCR. As shown in Fig. 3d–h, a significant reduction in all pro-inflammatory cytokines tested was observed in presence of GGEC as compared to control. In addition, the expression of iNOS and COX-2, was highly and dose-dependently down-regulated.

### Wound healing assay

**Fig. 4:**
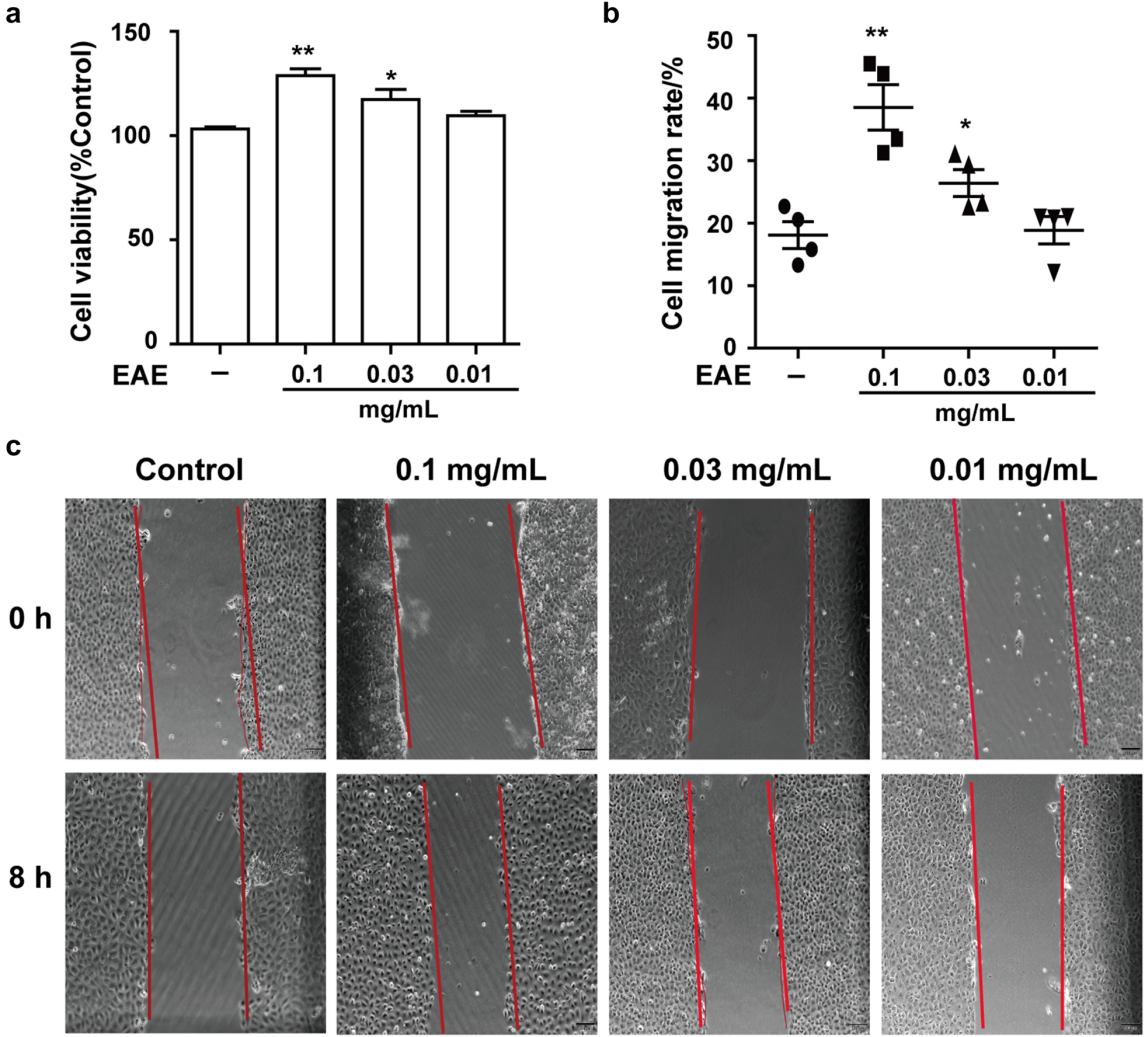
Effects of EAE on wound healing of IEC-6 cells. **a** The cytotoxic effect of EAE on cell viability in IEC-6 cells. **b** Wound healing activity on cell migration of EAE. **c** The representative image of the wound healing assay. Mean ± SD (n = 4 independent experiments). *p < 0.05, **p < 0.01 vs. the control group. EAE: galli gigeriae endothelium corneum ethyl acetate extract

In order to observe the effect of GGEC extract on the restoration of IEC-6 cells, the cell viability was performed firstly to select the optimal concentrations for further study. As shown in Fig. 4a, EAE at 0.1 mg/mL significantly increased the cell viability (132%). This result indicated that EAE had a positive effect on cell proliferation. Then the wound healing assay was taken to assess this effect. Compared with the control cells, IEC-6 cells treated with EAE (0.1 and 0.03 mg/mL) showed significantly enhanced wound healing after 8 h of incubation (Fig. 4b, c).

## Discussion

It is well recognized that epithelial cells covering the surface of the gastrointestinal tract are vital to the health functioning as a barrier [32]. The barrier confers a direct property of selective permeability to the intestine [4, 33]. TJs function as the physical intestinal barrier by regulating the ions, solutes, and water across the intestinal epithelium, protecting against extracellular substances such as antigens, organisms and xenobiotics [5, 12]. Numerous factors such as pro-inflammatory factors, LPS, and bacteria influence TJ homeostasis [34]. It was reported that TNF-α promoted TJ permeability and damaged barrier function due to decrease ZO-1 protein level and activation of the NF-κB pathway in Caco-2 cells [35]. In this study, we demostrated that EAE alleviated TNF-α induced the reduction of TEER and increase of FD-4 in paracellular permeability and suppressed the TNF-α-evoked down-regulated protein expression of ZO-1 and occludin.

Inflammatory bowel diseases (IBDs), including ulcerative colitis and Crohn’s disease, are well-known recurring and chronic inflammatory conditions of the intestinal epithelial cells [5, 7]. It was reported that excessive pro-inflammatory factors may destroy the intestinal barrier function and a dysfunctional barrier facilitated the passage of antigens through the epithelium [36], thereby eliciting an immune response leading to inflammation and subsequently various gastrointestinal disease symptoms [37]. Therefore, inhibition of pro-inflammatory cytokine secretion and improvement of intestinal barrier function are strategies for the prevention and treatment of IBD [38]. In this study, EAE effectively inhibited the mRNA expression of pro-inflammatory cytokines (IL-6 and IL-1β) in TNF-α-stimulated IEC-6 cells.

In the intestine, a large amount of pro-inflammatory factors was mainly produced by the activated macrophages [37]. Macrophages participate in and promote immune responses by inducing the expression of pro-inflammatory cytokines (TNF-α, IL-6, and IL-1β) when stimulated by the exogenous substances such as LPS. Our results showed that EAE reduced the production of pro-inflammatory cytokines (TNF-α, IL-6, and IL-1β) and inflammatory mediators (iNOS and COX-2) in LPS-stimulated RAW264.7 cells. The reduction of release of pro-inflammatory cytokines by EAE signified its potential beneficial role in intestinal epithelial barrier function.

It is crucial to retain the integrity of the epithelium for digesting food, absorbing nutrients and preventing harmful external agents from entering the body [5]. When the surface of the intestinal epithelium is damaged, IECs migrate to the injured region and proliferate to maintain homeostasis [35]. Therefore, improving the migration and proliferation of IECs is a promising therapeutic strategy for intestinal disorders [39]. This study aimed to investigate the influence of EAE on the migration and proliferation of IEC-6 cells. The wound healing assay results showed that treatment of EAE effectively promoted IEC-6 cells proliferation and migration.

It is reported that GGEC is rich in protein, amino acids and polysaccharide that contributed to its biological activities [8, 10]. However, the definite constituents still remain unclear. In this work, we explored the compounds in GGEC by HPLC-QTOF–MS/MS. There were 33 compounds including 12 isoflavones, 7 bile acids, 4 nucleic bases and nucleosides as well as 10 others that were systematically identified in EAE. This is the first time that BAs and isoflavones were tentatively identified in GGEC. Some studies suggested that bile acids are important regulators of epithelial integrity and might be the good targets for development of new candidates to modulate intestinal barrier function in diseases treatment [40]. It was reported that BAs, especially chenodeoxycholic acid (CDCA), could protect intestinal epithelial barrier in IPEC-J2 cells and mice via the FXR−MLCK signaling pathway [41, 42]. Studies have also shown that various flavonoids participate in the regulation of intestinal TJ barrier integrity [16]. However, whether the bile acids and isoflavones in GGEC were the main constituents that exerted the intestinal barrier protective effects remains further studies.

## Conclusions

In this study, we showed that EAE was mainly composed of isoflavones, cholic acids, amino acids and other molecules. It could ameliorate dysfunction of the intestinal epithelial barrier in IEC-6 cells via inhibiting the expression of the inflammatory mediators IL-6 and IL-1β, promoting epithelial proliferation and up-regulation of ZO-1 and Occludin protein expression. EAE also significantly reduced LPS-induced NO and mRNA expression levels of TNF-α, IL-1β, IL-6, iNOS and COX-2. The present findings demonstrated that GGEC extract exerted gastrointestinal barrier protective activities via strengthening the link between the cells and limiting inflammatory responses.

## Data Availability

The research data generated from this study is included within the article.

## Abbreviations

GGEC: Galli gigeriae endothelium corneum
EAE: GGEC ethyl acetate extract
FD: Functional dyspepsia
IECs: Intestinal epithelial cells
TJs: Tight junctions
IEC-6: Rat intestinal epithelial cells
DMSO: Dimethyl sulfoxide
HPLC-Q-TOF–MS: High-performance liquid chromatography coupled with quadrupole-time-of-flight high-definition mass spectrometry
ESI: Electrospray ionization
NO: Nitric oxide
IL-1β: Interleukin-1β
TNF-α: Tumor necrosis factor alpha
TEER: Transepithelial electrical resistance
iNOS: Inducible nitric oxide synthase
COX-2: Cyclooxygenase-2
FD-4: Fluorescein isothiocyanate–dextran, average mol wt 3000–5000
qRT-PCR: Real-time quantitative reverse transcription polymerase chain reaction
LPS: Lipopolysaccharide
IBDs: Inflammatory bowel diseases
CDCA: Chenodeoxycholic acid
